# Putative Key Role of Inositol Messengers in Endothelial Cells in Preeclampsia

**DOI:** 10.1155/2016/7695648

**Published:** 2016-09-21

**Authors:** Sirilaksana Kunjara, Patricia McLean, Laurens Rademacher, Thomas W. Rademacher, Fabiana Fascilla, Stefano Bettocchi, Marco Scioscia

**Affiliations:** ^1^Division of Biosciences, Research Department of Cell and Developmental Biology, University College London, London, UK; ^2^Emergex Vaccines Ltd, Abingdon, Oxfordshire, UK; ^3^Division of Infection and Immunity, University College London, London, UK; ^4^Department of Biomedical Sciences and Human Oncology (DIMO), II Unit of Obstetrics and Gynecology, University of Bari Aldo Moro, Bari, Italy; ^5^Department of Obstetrics and Gynecology, Sacro Cuore Don Calabria, Negrar, Verona, Italy

## Abstract

Immunological alterations, endothelial dysfunction, and insulin resistance characterize preeclampsia. Endothelial cells hold the key role in the pathogenesis of this disease. The signaling pathways mediating these biological abnormalities converge on PKB/Akt, an intracellular kinase regulating cell survival, proliferation, and metabolism. Inositol second messengers are involved in metabolic and cell signaling pathways and are highly expressed during preeclampsia. Intracellular action of these molecules is deeply affected by zinc, manganese, and calcium. To evaluate the pathophysiological significance, we present the response of the intracellular pathways of inositol phosphoglycans involved in cellular metabolism and propose a link with the disease.

## 1. Introduction

Immunological alterations, abnormal placental development, and endothelial cell dysfunction are widely accepted as major determinants for preeclampsia, a severe complication of human pregnancy that may be associated with poor maternal and fetal outcomes [1]. Preeclampsia is clinically defined as new hypertension and proteinuria developed in the second half of pregnancy. Other systemic alterations have been shown to be strictly linked to the pathophysiology of the disease, particularly inflammation, insulin resistance, and dyslipidemia [2, 3], which represent, along with hypertension, the main criteria for the diagnosis of the metabolic syndrome [4]. Several studies suggest that the metabolic syndrome contributes also to abnormal placental development and supports in a vicious cycle inflammation and endothelial dysfunction [5]. Mazar et al. [6] showed that increasing severity of the metabolic syndrome in pregnant women correlates independently with the development of preeclampsia, especially severe early-onset disease that is generally characterized by peculiar placental lesions [7]. Typical features of these alterations are an inadequate/incomplete trophoblast invasion of maternal spiral arteries [7] and acute atherosis [8]. Alterations of trophoblast invasion are mainly linked to failure of the extravillous trophoblast to adequately transform the uterine spiral arteries as a result of altered immunological response at the fetal-maternal interface [9, 10]. Uterine natural killer (uNK) cells seem to play a crucial role in the initial stages of spiral artery remodeling through vascular endothelial growth factor C (VEGF-C) [11]. VEGF-C expression in uNK cells is regulated by phosphoinositide 3-kinase (PI3-K)/Akt signaling pathway [12]. Furthermore, impaired invasion has also been hypothesized to be generated by abnormalities of the histiotrophic nutrition of trophoblast cells at early stages [13]. Acute atherosis is characterized by the presence of foam cells and lipid inclusions that resemble atherosclerotic lesions [8], with the important difference that atherosclerosis develops over decades while the lesions found in placental vessels in preeclampsia accumulate over a few months. These two aspects support a key role of the metabolic syndrome (inflammation and impaired glucose metabolism) in abnormal placental development.

At present, the correlation between endothelial dysfunction and metabolic syndrome in preeclampsia is still elusive. A growing body of evidence is emerging to show that the inositol second messenger system (myo-inositol and D-*chiro* inositol, two stereoisomers) is involved in metabolic and cell signaling pathways [14]. These molecules are precursors of important signaling molecules, including inositol phosphoglycans (IPG) that are also known as IPG-A and IPG-P, containing myo-inositol (MI) and D-*chiro* inositol (DCI), respectively. These second messengers mediate different actions of insulin: IPG-A stimulates lipogenesis, activates acetyl-CoA carboxylase, inhibits cAMP-dependent protein kinase, and modifies the activities of adenylate cyclase and cAMP-phosphodiesterase; IPG-P was shown to exert specific insulin-mimetic properties on the glycogen metabolism through the activation of pyruvate dehydrogenase phosphatase, glycogen synthase phosphatase, and glycerol-3-phosphate acyltransferase (IPG characteristics are summarized in Varela-Nieto et al., [15]). The two forms modulate insulin action and enhance insulin sensitivity [16]. D-*chiro* inositol phosphoglycans P-type (IPG-P) were shown to be highly increased in preeclampsia [17, 18].

Maternal clinical syndrome in preeclampsia (hypertension and proteinuria) derives from an imbalance of circulating angiogenic factors that results in maternal endothelial dysfunction [19]. We have recently proposed a potential convergence of intracellular pathways between angiogenic factors and inositol messengers on protein kinase B (PKB/Akt) [5]. PKB/Akt is a serine/threonine protein kinase that is activated by a variety of stimuli in endothelial cells, including multiple growth factors (VEGF, IGF-1, and HGF), estrogens, reactive oxygen species, mechanical stimuli, and drugs (i.e., statins). PKB/Akt is a well-known antiapoptotic factor, but it also regulates endothelial cell survival, migration, tube formation, and nitric oxide production [20]. Intracellular action of inositol messengers also depends on the concentrations of some elements like calcium and manganese that may promote or impair signaling transduction. In this article, we summarize the role of these elements and propose an overview of this intracellular system.

## 2. Regulation of the Pyruvate Dehydrogenase Complex (PDC) by Inositol Phosphoglycans

**Figure 1:**
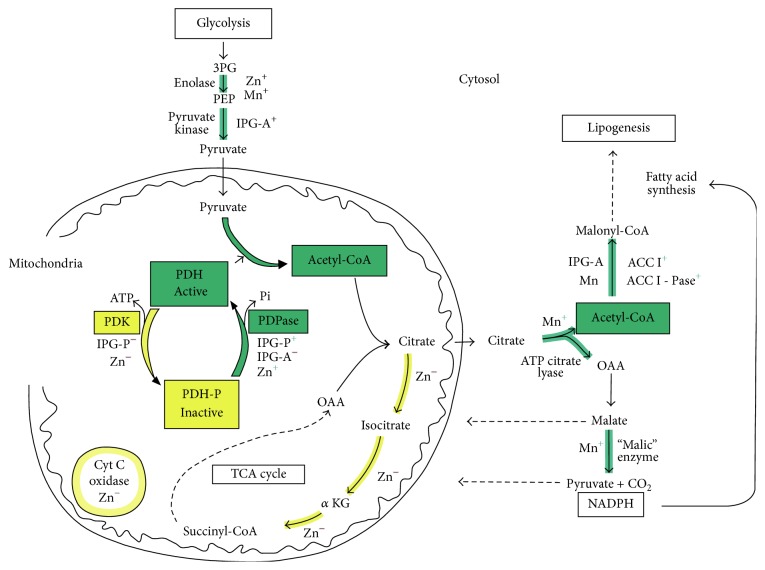
Aerobic glycolysis in the cytosol of cells produces pyruvate that enters the mitochondria where it is oxidized to acetyl-CoA by the pyruvate dehydrogenase complex (PDC). Inositol phosphoglycans (IPG) regulate the activation of PDC and, in its myo-inositol form (IPG-A), promote also lipogenesis in the cytosol. Zinc and manganese hold a key role in this system being involved in most steps as shown.

**Figure 2:**
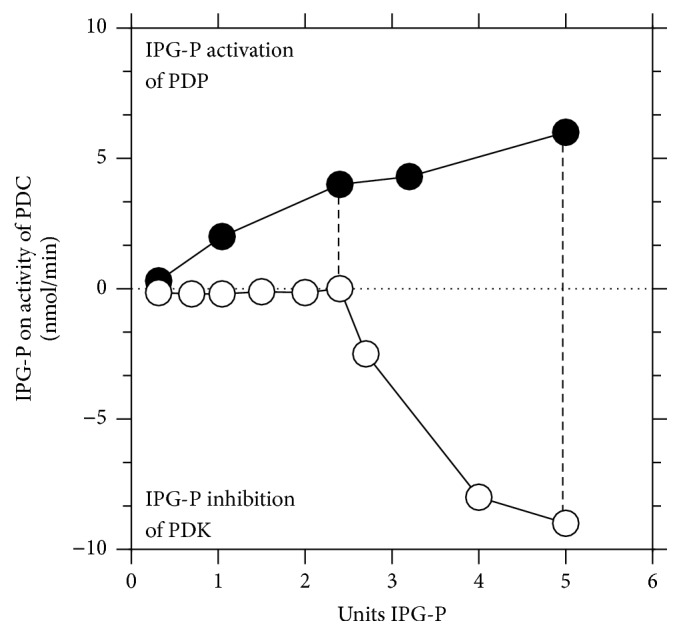
Inositol phosphoglycans P-type (IPG-P), which contains D-*chiro* inositol, can regulate the pyruvate dehydrogenase complex (PDC) by activating pyruvate dehydrogenase phosphatase (PDP) and inhibiting pyruvate dehydrogenase kinase (PDK) in a linear and sigmoid dose-dependent manner, respectively.

**Figure 3:**
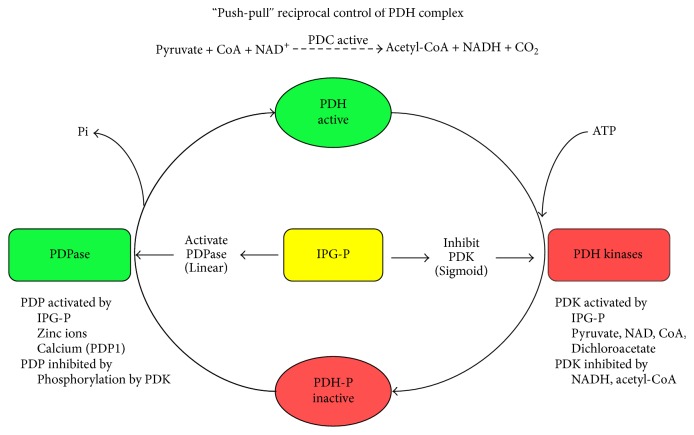
The reciprocal control of the pyruvate dehydrogenase complex has been shown in a previous article of ours [21] and is here represented in a scheme.

Reversible phosphorylation of proteins regulates many cell functions and abnormal phosphorylation can be associated with altered signaling leading to a variety of pathogenic states. Pyruvate dehydrogenase complex (PDC) is the key interface between glycolysis and the citric acid cycle and is crucial to the generation of ATP, acetyl-CoA, and NADH by mitochondria (Figure 1). The restoration of ATP following its loss during metabolic activity (or in the heart during ischemia) is of critical importance and subsequent ATP generation is dependent on PDC activity. PDC exists in dynamic equilibrium between dephosphorylated and phosphorylated forms (active and inactive, resp.) and the degree of phosphorylation is controlled by PDH phosphatases (PDP 1,2) and PDH kinases (PDK 1–4). PDK4 is selectively upregulated and PDP2 is downregulated in many tissues in response to starvation, diabetes, and insulin-resistant states [22]. We have previously shown that the putative insulin mediator inositol phosphoglycan P-type (IPG-P, containing D-*chiro* inositol) has a sigmoidal inhibitory action on PDK in addition to its known linear stimulation of PDP [21] (Figure 2). Thus, at critical intracellular levels of IPG-P, this sigmoidal/linear model could potentially amplify switchover from the inactive to active form of PDC, and a “push-pull” system that combined with the hormonal control of IPG-P indicates a powerful regulatory function via inhibition of PDK (Figure 3). The detection of bound zinc to the inositol phosphoglycans is of interest in relation to our reported effects of free zinc ions on PDP and PDH kinases [21] and also in the context of the cardioprotective effects of zinc on vascular ischemia-reperfusion injury [23].

## 3. Role of IPG Trace Metals in the Regulation of PDC and Channeling of Acetyl-CoA into Oxidative or Lipogenic Routes

Many factors need to be considered in order to understand the relationship between IPG-P and IPG-A types in the regulation of the PDH complex (PDC). These include the differing carbohydrate moieties and structures of the IPG and the binding affinities of their associated trace elements Mn^++^ and Zn^++^. In view of the number of enzymes and their compartmentation within biosynthetic pathways affected by these trace metals, a number of factors also need to be taken into consideration when extrapolating* in vitro* results to potential* in vivo* intracellular actions. Specifically, it is important to evaluate the free versus the total concentration of the metal ions in view of the presence of metal chelators in the assay system; we have to remember the fact that* in vitro* the PDP is in a soluble form and thus accessible to the effector molecules, either trace metal and/or IPG, in contrast to the* in vivo* situation where the PDK is associated with the mitochondrial membrane; and the* in vitro* system used to study effects on the PDP and PDK components of the PDC is not subject to the complex intracellular network of metal trafficking pathways [24, 25]. These authors emphasized not only the broad range of enzymes requiring metal ions for activity but also their diverse location. Luk et al. [24] stated the following: “the current paradigm is that metal ions are not free agents. Rather, these ions are under careful surveillance by systems designed to detoxify and sequester the metal or to escort the ion to its cognate site in a metalloprotein.”

It has been proposed that the effects of IPG-A and IPG-P containing zinc and manganese may be specifically linked to formation of acetyl groups via effects of zinc on the regulation of the PDC, that is, to the generation of acetyl groups via the tricarboxylic acid cycle [26, 27], or towards lipogenic pathways activated by manganese [28–30]. The data presented in Figure 2 suggests that Zn^++^ ions may be more significant in the regulation of PDC. Maximal activation of PDP is achieved with 0.01 *µ*M Zn^++^ but significant activation is still observed at a concentration of 0.003 *µ*M, a value commensurate with the measured content of zinc in IPG-P isolated from rat liver [21]. Interestingly, this latter value is poised on the steeply rising slope of activation; thus small changes in availability of Zn^++^ could have significant effects on PDP activity. The higher level of Zn^++^ required* in vitro* for the inhibition of PDK may well relate to the association of the enzyme with the mitochondrial membrane* in vivo* and the requirement for a transport system or catalytic chelators to locate the metal ion at the active site. We feel that these differences in inorganic ion concentration do not necessarily detract from the “push-pull” concept of the IPG-P in the control of the PDH complex but rather from limitations of the* in vitro* systems used.

**Figure 4:**
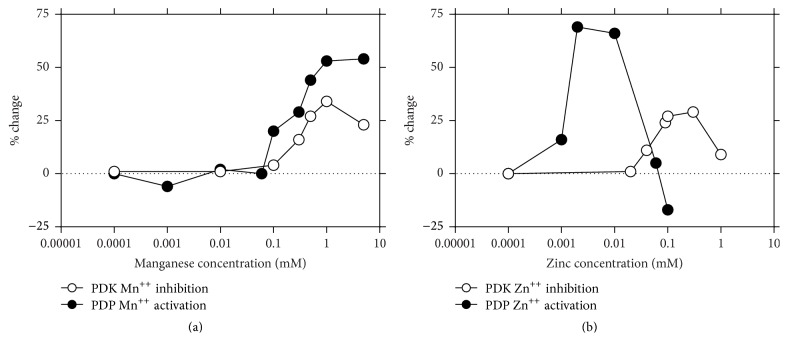
The activation of pyruvate dehydrogenase phosphatase (PDP) and the inhibition of pyruvate dehydrogenase kinase (PDK) are influenced by ion concentrations.

The hyperbolic curve of the effects of Zn^++^ on PDP (Figure 4(b)) falling steeply at concentrations above 0.01 mM may be of significance in relation to the inhibitory effects of IPG-A on the activation of PDP by IPG-P previously reported [31]. IPG-A has a 5-fold higher content of zinc relative to IPG-P and it is suggested that this level of zinc might reach a concentration commensurate with those on the descending arm of the bell shaped curve.

In contrast to the regulation by Zn^++^, the effect of Mn^++^ on the components of PDC is seen only at considerably higher concentrations (Figure 4(a)). It is notable that IPG-A contains a high content of manganese and this metal is also a known activator of lipogenesis and acetyl-CoA carboxylase [32]. It is suggested that the manganese content of the IPG may be related to the established effects of this trace metal on a range of enzymes linked to lipogenesis. Scorpio and Masoro [33] have shown that the acetyl-CoA carboxylase system is highly sensitive to Mn^++^ and that 50 *µ*M causes almost maximum activation, and approximately 50% maximal activation was shown at 25 *µ*M, a concentration commensurate with the manganese content of IPG-A. In addition, the activation of acetyl-CoA carboxylase by a manganese-dependent phosphatase has been reported [34].

In the present context, the known potent stimulatory effect of free zinc on lipogenesis [35] is considered in relation to the effect of this metal ion on enzymes linked to the formation of acetyl groups and direction of this metabolite to lipid synthesis. We propose that central to this role of zinc is its action as an activator of PDP and inhibitor of PDK thus promoting the conversion of pyruvate to acetyl-CoA. This resetting of the PDC to the active nonphosphorylated state and formation of acetyl-CoA may be enhanced by the effect of zinc on the activation of enolase in the glycolytic pathway [36] and by the regulation of the phosphorylation state of the insulin receptor [37] and thus downstream pathways favor the provision of pyruvate from glucose. The counter effect of zinc in depressing acetyl-CoA oxidation via inhibition of key enzymes of the tricarboxylic acid cycle including aconitase, NAD-linked isocitrate dehydrogenase, alpha-ketoglutarate dehydrogenase, succinate oxidation, and cytochrome c oxidase [26] could have the effect of both depressing ATP formation and preserving citrate for export to the cytosolic compartment and driving acetyl-CoA towards the lipogenic route. The existence of such metabolic pathways has increased interest in the insulin-mimetic actions of zinc complexes on adipocytes* in vitro* [38] and potential use in the treatment of type 2 diabetes and metabolic syndromes [39].

It is proposed that manganese, present in high concentrations in IPG-A type, could coordinate the conversion of acetyl-CoA to malonyl-CoA and lipid synthesis via manganese activation of ATP-citrate lyase, acetyl-CoA carboxylase 1, and acetyl-CoA carboxylase 1 phosphatase [34, 40]. Manganese is also an activator of “malic” enzyme [41] and thus the provision of NADPH for reductive synthesis and anaplerotic provision of pyruvate. It may be noted that both zinc and manganese are involved in the activation of enolase [36]. Additionally, manganese increases the stimulation by ATP of a putative insulin mediator from liver plasma cell membranes [24] and can largely overcome the regulatory feedback mechanisms of a high fat diet and increase lipogenic enzymes [28]. These observations are in accord with the reported effects of IPG-A on key enzymes of lipogenesis [32].

**Figure 5:**
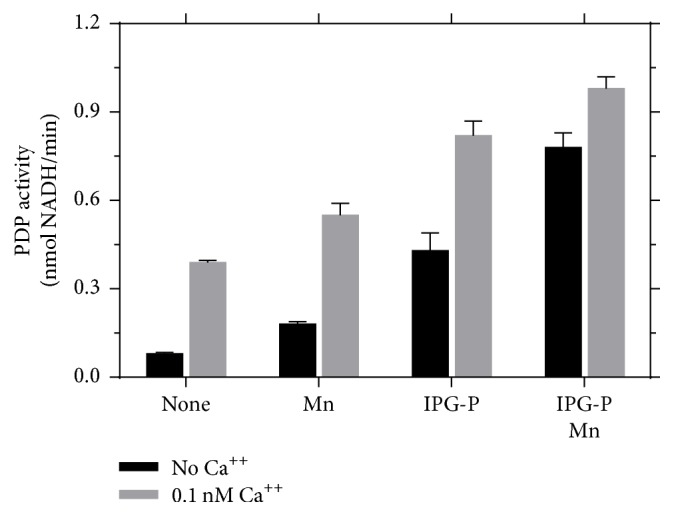
Pyruvate dehydrogenase phosphatase (PDP) can be activated in presence of calcium. This effect is boosted by inositol phosphoglycans P-type (IPG-P) and manganese.

PDP is divided into two isoforms that respond to insulin and IPG-P, where the isoform 1 requires calcium ions for the activation while the isoform 2 is insensitive to calcium [Roche]. PDP isoform 1 has been correlated with energy production in muscle and heart while PDP isoform 2 acts in starvation and diabetes as demonstrated in the animal model [42]. Measurements of the activity of PDP demonstrate that IPG-P is able to activate the enzyme on both isoforms (Figure 5) and they are optimally activated by the presence of Ca^++^ and Mn^++^ that act in synergy.

## 4. Discussion

To fully understand the potential role of these messengers in preeclampsia, a comparison with normal pregnancy has to be made. Healthy human pregnancy is characterized by an increasing insulin resistance throughout gestation [43]. Most pregnancies remain well compensated; others develop insulin-resistance related diseases like gestational diabetes and preeclampsia. The role of inositol messengers in insulin resistance is well known in nonpregnant subjects [44, 45] and growing evidence supports a definite role for these molecules in human pregnancy. Placental metabolism holds a pivotal role for the development and maintenance of a healthy pregnancy. Nestler et al. [46] showed that inositol messengers regulate insulin's steroidogenic actions in human placental cytotrophoblasts. In the recent years, inositol messengers were postulated to contribute to carbohydrate metabolism from early stages in human placenta when histiotrophic nutrition takes place [47]. At this time, glycogen and carbohydrate-rich secretions of the endometrial glands represent the main nutrient of trophoblast cells [48]. In fact, during placentation, the trophoblast invades the maternal endometrial glands and submucosal capillary network before reaching the spiral arteries. The first consequence of this is that the oxygen concentration within the intervillous space is relatively low compared to values during the second and third trimester [49] and this prevents a classical aerobic glycolysis (Wharburg metabolism). Inositol messengers were postulated to mediate this situation in the very first weeks of conception, promoting anaerobic metabolism of carbohydrates in human placenta [47]. Mechanisms underlying sugar metabolism through PDC complex have been reported above. Histological studies on healthy placental specimens collected in late first trimester failed to detect DCI in any structure (villi, stroma, and vessels) while a strong staining was found in term placentas [50, 51]. In fact, Kunjara et al. [52] reported a 25-fold increase of DCI concentration in healthy human placenta at term compared to normal liver (standard reference tissue for inositol messengers). It is interesting to highlight that a disrupted intracellular signal related to inositols takes place during preeclampsia. First reports of a relationship between inositol messengers and pregnancy complications were reported by Rademacher's group [52]. They reported with a threefold increase of DCI in placental specimens of preeclamptic mothers compared to healthy samples while no myo-inositol messenger was detected. Histological studies demonstrated a more intensive staining in villous stroma of preeclamptic placental specimens compared to gestation-matched controls [53]. Furthermore, a tendency towards a higher staining in samples of severe early-onset preeclampsia was reported. In fact, it has been demonstrated that insulin treatment of isolated membranes of insulin-sensitive tissues like normal human placental membranes and BC_3_H-1 myocytes results in the production of soluble DCI messengers [54, 55]. We confirmed a high release of DCI messengers after insulin incubation of fresh placental membranes from healthy pregnancy but found that there was no release of messengers after insulin stimulus in preeclamptic samples [56]. This may be explained by IRS-1 and IRS-2 inhibition due to serine-phosphorylation and subsequent impairment of downstream insulin signaling in preeclampsia [56]. This altered pathway prevents or impairs glycogen synthase (GS) that is normally activated via PI3K–PDK–Akt–GS kinase-3. Activated Akt also leads to membrane fusion of GLUT4 vesicles and promotes the action of mammalian target of rapamycin (mTOR) kinase that regulates many cell processes such as growth, proliferation, survival, protein synthesis, and gene transcription. Furthermore, DCI can be transported into mitochondria to activate PDH phosphatase, which in turn activates PDH.

Increased levels of DCI-phosphoglycan (IPG-P) were reported in many maternal and fetal fluids and tissues during preeclampsia (as reviewed by Scioscia et al., [57]). The longitudinal assessment of maternal urine specimens revealed significant increased excretion of DCI-phosphoglycans a few weeks before the onset of clinical preeclampsia [18, 58] and during active preeclampsia [59]. Inactivation of proteins downstream to insulin receptor can be induced by fatty acids, cellular stress, or diverse inflammatory cytokines, including TNF*α*, IL-1, or lipopolysaccharide [60]. According to our findings, the increased production of DCI-phosphoglycans during preeclampsia may be a compensatory effect to overcome IRS inactivation. Furthermore, hypertension in insulin-resistant states, including preeclampsia, is determined by inadequate vasodilation and paradoxical vasoconstriction through collateral signaling pathways (VEGF, IGF, and insulin) [60–64]. In a recent report, we have shown that insulin and VEGF signaling pathways related to vascular vasodilator/vasoconstrictor effects converge on PKB/Akt [5]. In fact, inhibition of the VEGF/Akt/eNOS pathway blocks VEGF-driven nitric oxide release and promotes vasoconstriction [65]. The activation of the insulin signaling in vascular cells through IRS-1/PI-3K/Akt results in eNOS phosphorylation on Ser1177, leading to enhanced nitric oxide production [66]. Along with this hypothesis, insulin-dependent activation of eNOS through the hypoxia inducible factor 1 was shown to be linked to subsequent increased expression of VEGF-A [67].

PDP activity is strictly related to ion concentrations in mitochondria. The presence of Ca^++^, Mn^++^, and Zn^++^ strengthens the response to IPG-P. This may be of importance when increased workload and adrenergic stimulation occurs, for instance, in muscle and heart cells under stress during hyperglycemia and starvation.

Certainly, the interaction between intracellular pathways related to metabolism and vascular alterations is not fully explained yet. We argue that a large part of the pathophysiology of preeclampsia may be based on this interaction given the observation that both aspects have been described in preeclampsia and long-term alterations (higher risk of metabolic syndrome and cardiovascular disease) can certainly represent the final expression of these disrupted cross talk pathways. Whether inositol second messengers are among the key molecules in this process as it seems nowadays has been shown in detail. Many points have been already demonstrated while minor linking aspects should be investigated.

Characterization of the intracellular action of IPG-P in the context of a metabolic disease like preeclampsia helps in understanding the underlying biochemical mechanisms that occur in placenta and endothelial cells. This may help in the characterization of the link between alterations reported in human placenta and the endothelial reaction that occurs in this disease. Indeed, these aspects have to be fully elucidated in human tissues of women with preeclampsia, so further studies are warranted. On the other hand, this may lead to pharmacological interventions to support and/or prevent anomalies that lead to the development of preeclampsia.
